# Determinants of the onset and prognosis of the post-COVID-19 condition: a 2-year prospective observational cohort study

**DOI:** 10.1016/j.lanepe.2023.100724

**Published:** 2023-09-05

**Authors:** Lourdes Mateu, Cristian Tebe, Cora Loste, José Ramón Santos, Gemma Lladós, Cristina López, Sergio España-Cueto, Ruth Toledo, Marta Font, Anna Chamorro, Francisco Muñoz-López, Maria Nevot, Nuria Vallejo, Albert Teis, Jordi Puig, Carmina R. Fumaz, José A. Muñoz-Moreno, Anna Prats, Carla Estany-Quera, Roser Coll-Fernández, Cristina Herrero, Patricia Casares, Ana Garcia, Bonaventura Clotet, Roger Paredes, Marta Massanella

**Affiliations:** a Department of Infectious Diseases, Hospital Germans Trias i Pujol, Badalona, Catalonia, Spain; b Fundació Lluita Contra les Infeccions, Badalona, Catalonia, Spain; c Universitat Autònoma de Barcelona, Catalonia, Spain; d Universitat de Vic – UCC, Vic, Catalonia, Spain; e REICOP, Spain; f Biostatistics Unit, Hospital Germans Trias i Pujol, Institut de Recerca Germans Trias i Pujol, Can Ruti Campus, Badalona, Catalonia, Spain; g IrsiCaixa AIDS Research Institute, Germans Trias i Pujol Research Institute (IGTP), Can Ruti Campus, Badalona, Catalonia, Spain; h Cardiology Department, Hospital Germans Trias i Pujol, Badalona, Catalonia, Spain; i Department of Rehabilitation, Hospital Germans Trias i Pujol, Can Ruti Campus, Badalona, Catalonia, Spain; j CIBER Infectious Diseases (CIBERINFEC), Institute of Health Carlos III (ISCIII), Madrid, Spain; k Center for Global Health and Diseases, Department of Pathology, Case Western Reserve University School of Medicine, Cleveland, OH, USA

**Keywords:** COVID-19, Post-COVID-19 condition, Long COVID-19

## Abstract

**Background:**

At least 5–10% of subjects surviving COVID-19 develop the post-COVID-19 condition (PCC) or “Long COVID”. The clinical presentation of PCC is heterogeneous, its pathogenesis is being deciphered, and objective, validated biomarkers are lacking. It is unknown if PCC is a single entity or a heterogeneous syndrome with overlapping pathophysiological basis. The large US RECOVER study identified four clusters of subjects with PCC according to their presenting symptoms. However, the long-term clinical implications of PCC remain unknown.

**Methods:**

We conducted a 2-year prospective cohort study of subjects surviving COVID-19, including individuals fulfilling the WHO PCC definition and subjects with full clinical recovery. We systematically collected post-COVID-19 symptoms using prespecified questionnaires and performed additional diagnostic imaging tests when needed. Factors associated with PCC were identified and modelled using logistic regression. Unsupervised clustering analysis was used to group subjects with PCC according to their presenting symptoms. Factors associated with PCC recovery were modelled using a direct acyclic graph approach.

**Findings:**

The study included 548 individuals, 341 with PCC, followed for a median of 23 months (IQR 16.5–23.5), and 207 subjects fully recovered. In the model with the best fit, subjects who were male and had tertiary studies were less likely to develop PCC, whereas a history of headache, or presence of tachycardia, fatigue, neurocognitive and neurosensitive complaints and dyspnea at COVID-19 diagnosis predicted the development of PCC. The cluster analysis revealed the presence of three symptom clusters with an additive number of symptoms. Only 26 subjects (7.6%) recovered from PCC during follow-up; almost all of them (n = 24) belonged to the less symptomatic cluster A, dominated mainly by fatigue. Recovery from PCC was more likely in subjects who were male, required ICU admission, or had cardiovascular comorbidities, hyporexia and/or smell/taste alterations during acute COVID-19. Subjects presenting with muscle pain, impaired attention, dyspnea, or tachycardia, conversely, were less likely to recover from PCC.

**Interpretation:**

Preexisting medical and socioeconomic factors, as well as acute COVID-19 symptoms, are associated with the development of and recovery from the PCC. Recovery is extremely rare during the first 2 years, posing a major challenge to healthcare systems.

**Funding:**

Fundació Lluita contra les Infeccions.

**Research in context**

**Evidence before this study**

We searched PubMed for observational studies investigating the characteristics and outcomes of patients with post-COVID-19 condition (PCC). The key terms “long COVID” or “post-COVID-19 condition” retrieved more than one thousand articles. However, only 23 corresponded to observational studies. Most of these studies were either retrospective analyses of electronic health records, survey- or phone-based studies of self-reported symptoms, or short series of patients who attended face-to-face visits. Two studies, conducted in the UK and Ireland, prospectively assessed patients with PCC after hospital discharge. One large crossectional study in the US identified four clusters of subjects with PCC according to their presenting symptoms and proposed an operational definition based on a symptom-scoring system. We did not find prospective studies investigating the evolution of PCC in face-to-face visits for follow-up periods of at least one year in adults. One study conducted a prospective clinical follow-up of a large children cohort, identifying risk factors and recovery rates of PCC in the pediatric population. Regardless of the study design, six articles found different phenotypes of PCC based on unsupervised clustering of symptoms. The long-term clinical implications of PCC, in particular the rate of recovery from this syndrome, remain unknown.

**Added value of this study**

In our study, we found that preexisting medical conditions, including several comorbidities, socioeconomic factors like the educational level, as well as specific symptoms during acute COVID-19 presentation, predicted both the development of and recovery from the PCC. In concordance with the RECOVER study, individuals presented with sub-syndromic clusters characterized by accumulation of overlapping symptoms rather than by exclusive syndromic profiles. Of note, recovery from PCC occurred in a minority of subjects during the first 2 years.

**Implications of all the available evidence**

Although the PCC has a heterogeneous presentation at the individual level, the pattern of the observed subphenotypes likely reflects the additive severity of a single, multisystemic, multifaceted post-viral disease rather than different pathogenically-independent subsyndromes. The initial COVID-19 clinical presentation, together with the comorbidity background and educational level of the patient, are associated with both the risk of onset and recovery from the PCC. Unfortunately, the small chances of recovering from PCC during the first 2 years underscore that, as long as SARS-CoV-2 transmissions continue and few people are cured, subjects with PCC will continue to accumulate. European healthcare systems must be prepared to absorb and manage such demand. Novel objective biomarkers, deeper pathophysiological insights and innovative therapies are urgently needed to prevent and cure PCC or, at least, mitigate its effects and its public health impact.

## Introduction

At least 5–10% of COVID-19 survivors develop a post-viral syndrome known as post-COVID-19 condition (PCC), post-acute COVID-19 sequelae (PACS) or “Long COVID”.^1^, ^2^, ^3^ This syndrome includes a variety of long-lasting, debilitating symptoms and medical conditions, which often lead to physical, social and psychological disability, and have a severe impact on patients’ quality of life.^4^, ^5^, ^6^, ^7^

Uncertainties around the pathophysiology of this syndrome, the lack of effective treatments, and the absence of validated biomarkers, force PCC to be identified and managed using clinical definitions that are useful but imprecise.^8^, ^9^, ^10^, ^11^, ^12^ It is unknown if PCC is a single entity or, instead, a heterogeneous composite of subsyndromes with an independent pathophysiological basis. In a recent large crossectional evaluation, the US RECOVER study^13^ proposed an operational definition of the PACS using a symptom score grading system. They also identified four clusters of subjects according to their presenting symptoms, driven, respectively, by smell/taste alterations, post-exertional malaise, brain fog, and palpitations and gastrointestinal symptoms. There was considerable overlap and progressive accumulation of symptoms among the four clusters. Symptom collection was self-reported by patients. Importantly, the long-term clinical implications of such clusters, in particular their chance to recover from PCC, remain unknown.

We sought to systematically characterize the clinical presentation and 2-year evolution of PCC, including the presence of different subsyndromes and factors associated with PCC onset and recovery, in a well-established European prospective cohort of COVID-19 survivors. Evaluations included systematic symptom collection using prespecified questionnaires, physical examination, and additional diagnostic imaging tests as needed.

## Methods

### Study design and population

This was an observational prospective cohort study of COVID-19 survivors who visited the Long COVID Unit of the Department of Infectious Diseases, Hospital Germans Trias i Pujol, Badalona, Spain. This is the largest monographic Long COVID Unit in Spain. It provides multidisciplinary care by physicians, nurses and psychologists to >1200 subjects with PCC, mainly from Catalonia but also from other Spanish regions. All study participants were included in a prospective cohort study of individuals exposed to SARS-CoV-2 (KING Cohort, HUGTIP/PI-20-217)^14^^,^^15^ between May 01, 2020 and February 17, 2022.

The diagnosis of PCC was defined based on the WHO criteria as the presence of persistent SARS-CoV-2-related symptoms (either relapsing or uninterrupted) at least three months from the onset of a COVID-19 episode.^12^ Symptoms related to PCC were considered to be those of new onset, or previously reported but with a significant worsening, that could not be attributed to other causes. The acute COVID-19 episode was confirmed by nasopharyngeal SARS-CoV-2 PCR, Lateral Flow Rapid Test, or serology. In subjects with PCC, we also accepted a clinical diagnosis of acute COVID-19. This was because, in Spain, as in many other regions in the World, subjects with COVID-19 who did not require hospitalization during the first SARS-CoV-2 epidemic wave did not have access to proper SARS-CoV-2 testing during the acute episode. Yet, they developed identical PCC symptoms to those with a microbiological diagnosis and are also followed in our Long COVID Unit. A comprehensive list of symptoms and functional scales were systematically collected using prespecified questionnaires (Supplementary Appendix) by the study physicians and nurses during follow-up face-to-face visits. To explore the short-term impact of SARS-CoV-2 vaccination in PCC symptoms, participants were asked if vaccination improved or worsened, either transiently (within 1 month after vaccination) or sustainedly (≥1 month) their PCC-associated symptoms, relative to their overall condition during the month previous to the vaccine administration.

The independent ethics committee of the Germans Trias i Pujol Hospital approved the study protocol (PI-20-217). All procedures were conducted according to good clinical practices and the General Data Protection Regulation 2016/679 on data protection and privacy for all individuals within the European Union. All study participants provided signed informed consent to participate in the study.

### Variables and data sources

All study data were collected and managed using a specifically designed REDCap (Research Electronic Data Capture) electronic case report form hosted at the Hospital Germans Trias i Pujol.^16^ The baseline visit was the earliest post-acute COVID-19 visit available within the KING Cohort. Subjects were visited at months 3, 6, 12, 18, and 24 from the date of acute COVID-19 diagnosis.

At baseline, we collected demographic data, comorbidities present at acute COVID-19 onset, and the characteristics of the acute COVID-19 episode, including the date, microbiologic test used to diagnose COVID-19, need for hospitalization, events that occurred during hospitalization (i.e., admission to ICU and need for mechanical ventilation), treatment received and diagnostic imaging tests. Data on the SARS-CoV-2 variant infecting each participant was inferred from the dominant circulating variant in Catalonia (Spain) at the time of infection according to GISAID (www.gisaid.org). The educational level of study participants was categorized in 4 groups: Less-than-basic education, primary education, secondary education, and tertiary education or higher. The latter category included post-secondary non-tertiary education, short-cycle tertiary education, Bachelor's, Master's or Doctoral degrees, or equivalent levels. Most variables were collected without any missing data, but for some patients, certain variables could not be retrieved from the previous clinical records (Supplementary Table S1, Supplementary Appendix).

We interviewed the patient about the presence of persistent symptoms, their type of onset (acute, gradual), and their clinical course (i.e., continuous or relapsing-remitting) using structured questionnaires (Supplementary Appendix). ‘Persistent symptoms’ were defined as COVID-19-related symptoms reported between 3 and 24 months after the acute episode and lasting at least two months, in line with the WHO PCC definition.^5^ In each follow-up visit, study participants were reinterrogated about the persistence of symptoms using standardized questionnaires. All patients with anginal chest pain were referred to the cardiology service for examination. Computerized tomography (CT) or magnetic resonance imaging (MRI) scans were performed as required per the cardiology evaluation.

A patient with PCC was considered ‘recovered from PCC’ when all persistent symptoms remitted for at least three consecutive months. Subjects with symptoms before PCC diagnosis were considered recovered when they returned to their baseline status prior to SARS-CoV-2 infection.

### Statistical analysis

The sociodemographic and clinical characteristics of study participants were described using frequencies and percentages over available data and means (standard deviation [SD]). Missing data were not imputed and were ruled out from the analysis.

Pre-existing conditions at COVID-19 diagnosis and symptoms at COVID-19 onset were analysed to assess their association with PCC development using a logistic regression model, with effects expressed as odds ratios with 95% confidence intervals. Two logistic regression models were then fitted for each set of factors. A combined strategy of statistician and clinician criteria was used for variable selection. We performed 250 bootstrap backward selections^17^ using the Akaike information criterion to identify those variables retained in more than 80% of the models as candidates for the final model. Following this first selection round, the final set of factors to be included in the models was discussed and evaluated by the clinicians.

We used hierarchical clustering analysis to group patients with PCC based on their presenting symptoms. A Gower’s distance was used to construct a dissimilarity matrix that accounts for the presence of both continuous and categorical variables. The optimal number of clusters was determined using an elbow plot. Internal validation was performed using silhouette scores to evaluate the similarity of patients within each cluster and the dissimilarity of patients between clusters. Finally, we conducted a detailed description of the clinical profile of the patients included in each cluster.

To investigate the role of different factors explaining PCC resolution, a direct acyclic graph (DAG)^18^ was constructed by representing causal assumptions based on previous literature and the clinical expertise of the research team managing patients with PCC. To assess the expected association and direction of these factors, a log-binomial regression model was estimated with remission symptoms as the dependent variable. The relative risk ratio with its 95% confidence interval (CI) were reported, and model conditions were assessed. The software R^19^ version 4.2.1 and its packages compareGroups,^20^ cluster,^21^ and performance^22^ were used for the statistical analysis.

### Role of the funding source

The study was funded through fundraising campaigns by the not-for-profit foundation *Fundació Lluita contra les Infeccions*, including the “yomecorono.org” and *Gala contra les Infeccions*, Editions 2021 and 2022. None of the funding sources was involved in the study design, data collection, data analysis, result interpretation, or writing of the report.

## Results

### Subjects’ characteristics

The study included 548 participants: 341 (62.2%) with and 207 (37.8%) without PCC (Supplementary Figure S1). The characteristics of both groups are summarized in Table 1, and Supplementary Table S2 and Supplementary Figure S2.

**Table 1:** Subjects’ characteristics at baseline.

	PCC N = 341	No PCC N = 207
Demographic		
Female, N (%)	238 (69.8)	93 (44.9)
Age (years), mean (SD)	47.9 (12.2)	48.7 (16.2)
Ethnicity, N (%)		
Caucasian/white	294 (86.2)	139 (88.5)
Healthcare worker, N (%)	85 (24.9)	60 (37.3)
Study level		
Below basic	2 (0.63)	1 (0.68)
Primary	43 (13.7)	20 (13.6)
Secondary	94 (29.8)	20 (13.6)
Tertiary or higher	176 (55.9)	106 (72.1)
Clinical		
Body mass index (Kg/m^2^), mean (SD)	26.7 (5.7)	26.0 (4.68)
Body mass index group, N (%)		
<18.5: underweight	9 (2.6)	6 (4.3)
18.5–24.9: healthy weight	128 (37.5)	54 (38.6)
25–29.9: overweight	105 (30.8)	58 (41.4)
30–34.9: Class I obesity	43 (12.6)	17 (12.1)
35–39.9: Class II obesity	25 (7.3)	3 (2.1)
40–49.9: Class III obesity (morbid)	9 (2.6)	2 (1.4)
Regular physical activity, n (%)	193 (56.6)	112 (79.4)
Comorbidities^a^, N (%)	308 (90.3)	155 (74.9)
Acute COVID-19 episode		
Hospitalized, N (%)	130 (38.1)	94 (45.4)
Required intensive care, N (%)	16 (4.7)	15 (7.2)
Required oxygen during hospitalization, N (%)		
No oxygen	28 (8.2)	28 (13.5)
Low flow oxygen	65 (19.0)	47 (22.7)
High flow/mechanical ventilation	31 (9.1)	15 (7.2)
Unknown	6 (1.7)	4 (1.9)
Diagnosis, N (%)		
Nasopharyngeal SARS-CoV-2 PCR	201 (58.9)	154 (74.4)
Lateral Flow Rapid Test	19 (5.6)	10 (4.8)
Serology	61 (17.9)	40 (19.3)
Clinical	60 (17.6)	3 (1.4)
SARS-CoV-2 variants, N (%)		
Ancestral	318 (93.3)	185 (89.4)
Alpha	23 (6.7)	20 (9.7)
Delta	0 (0)	2 (1.0)
SARS-CoV-2 vaccination, N (%)		
Previous to SARS-CoV-2 infection	2 (0.6)	10 (4.8)
After infection	299 (87.7)	144 (69.5)
Not vaccinated	40 (11.7)	53 (25.6)
Number of SARS-CoV-2 vaccine doses^b^, N (%)		
1 dose	75 (24.1)	33 (21.4)
2 doses	136 (45.2)	85 (55.2)
3 doses	90 (29.9)	36 (23.4)

A comprehensive list of comorbidities is shown in the Supplementary Table S1.

a PCC, post-COVID-19 condition.

b Number of vaccine doses among vaccinated.

Subjects with PCC had their baseline visit conducted in a median of 3.7 months (IQR 0.3–7.6) after the acute COVID-19 episode, and were followed for a median of 23 months (IQR 16.5–23.5) thereafter. They were most frequently middle-aged women with comorbidities (Table 1 and Supplementary Table S1), namely any type of allergy (31.4%), obesity (24.8%), dyslipidemia (24.0%), and hypertension (19.6%). Of the 238 women included in this study, 84 (35.3%) had menopause. Most acute COVID-19 episodes had been mild. Only 130 (38.1%) subjects were hospitalized during acute COVID-19, and only 16 (4.7%) required intensive care. In comparison, subjects without PCC were more frequently male (55.1%), healthcare workers (37.3%) with tertiary studies (72.1%), and had required hospitalization (45.4%).

Most individuals were first infected with SARS-CoV-2 during the pre-Omicron era, and virtually none of them had been immunized before the PCC diagnosis: only 2 subjects had received a complete 2-shot vaccine regimen 44 and 10 days before acute COVID-19, respectively; 7 were vaccinated between the acute infection and the PCC diagnosis (median time from acute infection was 2.2 months; IQR 0.6–2.4); the remaining individuals were vaccinated after the PCC diagnosis, a median of 12.6 months (IQR 9.4–14.8) after the acute COVID-19 episode.

### PCC clinical presentation

The symptom profile at COVID-19 onset of subjects with and without PCC is shown in Table 2. The most frequent (>50%) presenting symptoms in subjects later developing PCC were fatigue, dyspnea, neurocognitive complaints, headache, arthralgia, cough, diarrhea, chest pain, low-grade fever, myalgia, fever, smell alterations, tachycardia, and hair loss. Compared to those with PCC, subjects who did not develop PCC were less likely to report dyspnea, neurocognitive complaints, chest pain, myalgia, tachycardia or abdominal pain at COVID-19 onset. The most frequent persistent symptoms in subjects with PCC were fatigue, joint and muscle pain, dyspnea, headache, neurocognitive complaints, cough, chest pain, tachycardia, and diarrhea (Supplementary Figure S2).

**Table 2:** Symptom profile at COVID-19 onset.

	PCC N = 341 (%)	No PCC N = 207 (%)
Fatigue	317 (93.0)	135 (65.2)
Dyspnea	262 (76.8)	55 (26.6)
Neurocognitive complaints	247 (72.4)	23 (11.1)
Headache	240 (70.4)	105 (50.7)
Arthralgia	224 (65.7)	82 (39.6)
Cough	220 (64.5)	118 (57.0)
Diarrhea	198 (58.1)	71 (34.3)
Chest pain	186 (54.5)	23 (11.1)
Low grade fever	185 (54.3)	84 (40.6)
Myalgia	183 (53.7)	34 (16.4)
Fever	180 (52.8)	117 (56.5)
Smell alterations	174 (51.0)	77 (37.2)
Tachycardia	170 (49.9)	15 (7.2)
Hair loss	166 (48.7)	24 (11.6)
Taste alterations	160 (46.9)	74 (35.7)
Neurosensitive alterations	153 (44.9)	9 (4.3)
Skin alterations	132 (38.7)	20 (9.7)
Insomnia	99 (29.0)	3 (1.4)
Mucosal dryness	86 (25.2)	4 (1.9)
Weight loss	83 (24.3)	46 (22.2)
Dizziness	79 (23.2)	5 (2.4)
Abdominal pain	78 (22.9)	6 (2.9)
Visual alterations	76 (22.3)	2 (1.0)
Dysphonia	67 (19.6)	1 (0.5)
Sore throat	64 (18.8)	13 (6.3)
Dysphagia	64 (18.8)	5 (2.4)
Expectoration	62 (18.2)	33 (15.9)
Nausea	59 (17.3)	11 (5.3)
Sensation of dystermia	58 (17.0)	14 (6.8)
Constipation	58 (17.0)	10 (4.8)
Tinnitus	49 (14.4)	1 (0.5)
Hiporexia	36 (10.6)	13 (6.3)
Anorexia	34 (10,0)	11 (5.3)
Blood pressure alterations	22 (6.4)	3 (1.4)
Diaphoresis	19 (5.6)	4 (1.9)
Otalgia	10 (2.9)	2 (1.0)
Specific neurocognitive complaints		
Attention/concentration deficit	195 (57.2)	20 (9.7)
Memory loss	184 (54.0)	10 (4.8)
Difficulty in planning	116 (34.0)	9 (4.3)
Brain fog	43 (12.6)	0 (0.0)
Nominal aphasia	18 (5.3)	0 (0.0)
Writing/reading difficulty	10 (2.9)	0 (0.0)
Bradypsychia	7 (2.0)	1 (0.5)
Disorientation	5 (1.5)	1 (0.5)
Bradylalia	4 (1.2)	0 (0.0)

**Fig. 1:**
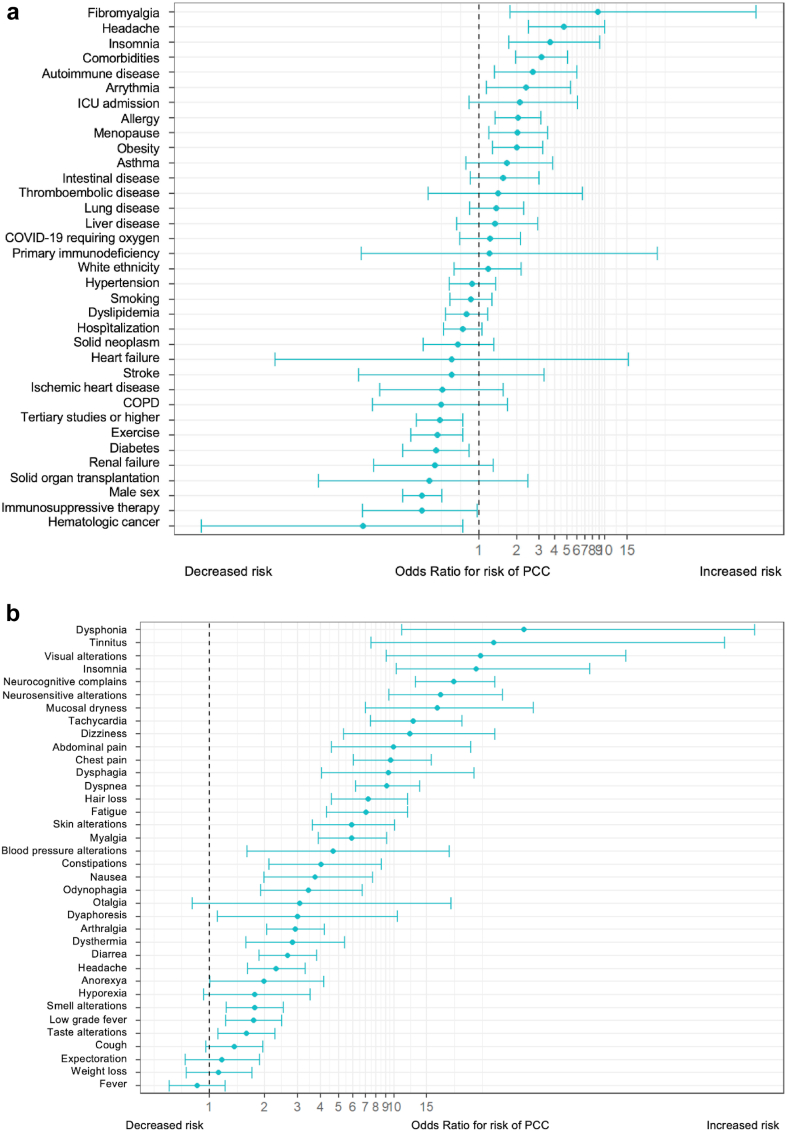
**Factors associated with the post-COVID-19 condition.** (a) Pre-existing conditions (odds ratio with 95% confidence interval; univariate logistic regression model). (b) Symptoms at COVID-19 diagnosis (odds ratio with 95% confidence interval; univariate logistic regression model).

Preexisting conditions associated with the PCC (Fig. 1) included fibromyalgia, history of headache or insomnia, comorbidities, autoimmune diseases and prior arrythmia episodes. A plethora of symptoms at COVID-19 onset were associated with an increased risk of PCC. Those with the highest magnitude of association were dysphonia, tinnitus, visual alterations, insomnia, neurocognitive complaints, neurosensitive alterations, mucosal dryness, tachycardia, dizziness, and abdominal and chest pain.

The logistic regression model of PCC risk with the best fit (AUC 0.931, 95% CI: 0.908–0.954) included pre-existing conditions plus symptoms at presentation (Table 3). According to this model, subjects who were male (OR 0.22, 95% CI: 0.07–0.64) and had tertiary studies or higher (OR 0.16, 95% CI: 0.05–0.43) were less likely to develop PCC, whereas those who had a history of headache (OR 5.18, 95% CI: 1.75–17.28), or presented with tachycardia (OR 4.93, 95% CI: 2.31–11.08), fatigue (OR 3.92, 95% CI: 1.70–9.71), neurocognitive (OR 6.75, 95% CI: 3.58–13.15) or neurosensitive (OR 7.73, 95% CI: 3.27–20.29) complaints and dyspnea (OR 4.32, 95% CI: 2.39–7.94) at COVID-19 onset were more likely to develop the PCC.

**Table 3:** Regression models for the post-COVID-19 condition.

Predictors	Model 1 (only including pre-existing conditions) n = 462 Area under the curve: 0.766 (0.723–0.809)				Model 2 (pre-existing conditions + presenting symptoms) n = 462 Area under the curve: 0.931 (0.908–0.954)			
	Odds ratio	Std. error	95% CI	p-value	Odds ratio	Std. error	95% CI	p-value
(Intercept)	9.66	4.06	4.52–24.00	<0.001	0.23	0.14	0.07–0.74	0.012
Male sex	0.13	0.06	0.05–0.31	<0.001	0.22	0.12	0.07–0.64	0.007
Tertiary studies or higher	0.14	0.06	0.06–0.32	<0.001	0.16	0.08	0.05–0.43	0.001
Diabetes	0.32	0.14	0.13–0.73	0.008				
Autoimmune disease	3.83	2.12	1.42–12.99	0.015				
Arrythmia	4.00	2.49	1.33–16.26	0.026				
Headache	3.31	1.45	1.48–8.45	0.006	5.18	3.00	1.75–17.28	0.005
Immunosuppressive treatment	0.09	0.08	0.02–0.48	0.006				
Insomnia	2.55	1.39	0.94–8.25	0.084				
Allergy	1.58	0.41	0.96–2.63	0.076				
Male sex × tertiary studies or higher	4.39	2.39	1.57–13.41	0.007	5.33	3.61	1.45–20.95	0.013
Tachycardia					4.93	1.96	2.31–11.08	<0.001
Fatigue					3.92	1.74	1.70–9.71	0.002
Neurocognitive complaints					6.75	2.23	3.58–13.15	<0.001
Neurosensitive complaints					7.73	3.56	3.27–20.29	<0.001
Dyspnea					4.32	1.32	2.39–7.94	<0.001

### PCC subsyndromes

**Fig. 2:**
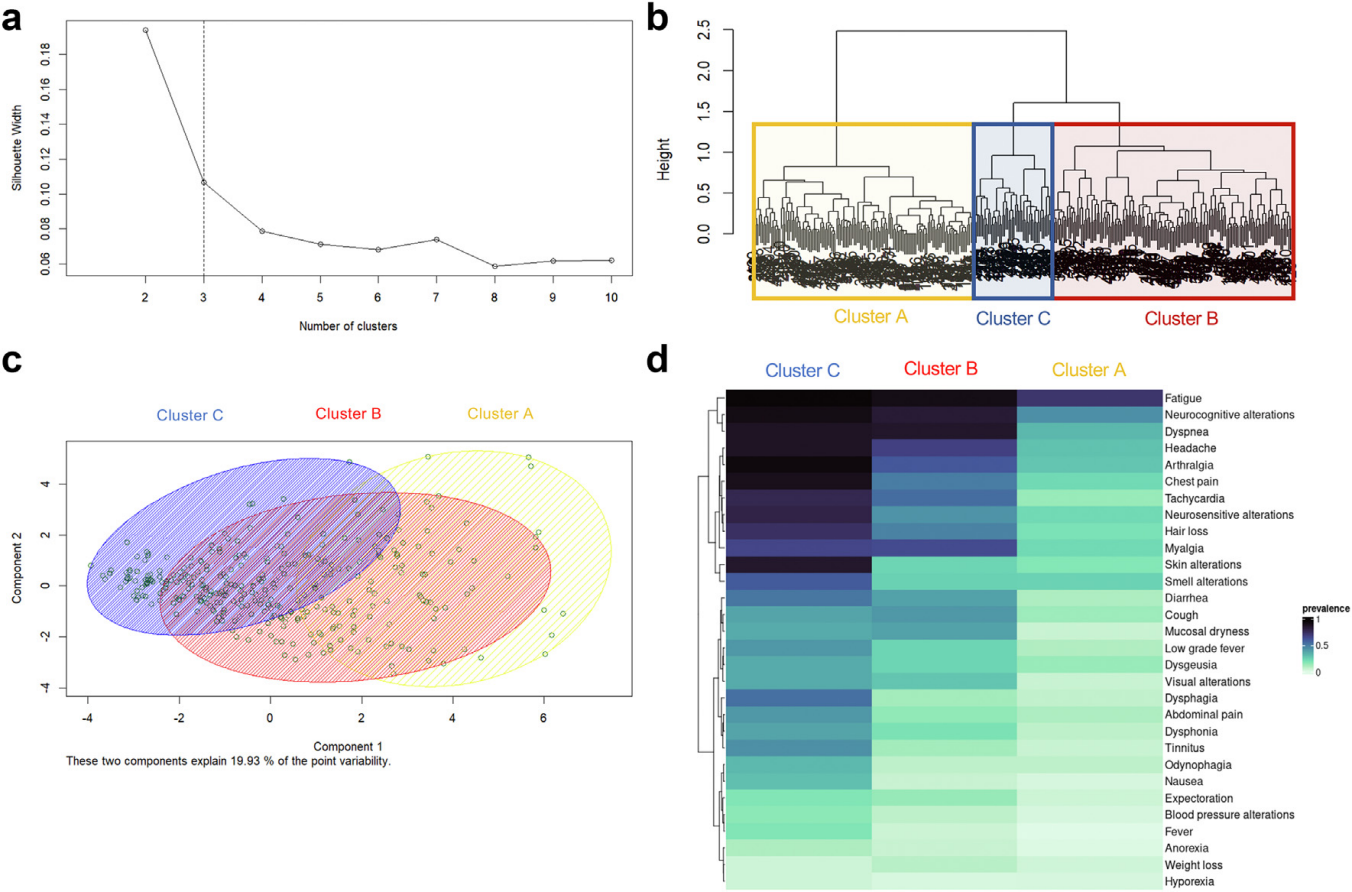
**Post-COVID-19 condition clusters.** (a) Silhouette plot suggesting a cut-off point on 3 clusters. (b) Dendogram of hierarchical cluster analysis on a set of dissimilarities using the Daisy method for mixed types of variables and the Gower metric. (c) 2-dimensional clustering plot, where each subject is represented by the principal components, and an ellipse is drawn around each cluster. (d) Heat map of subjects according to cluster membership and symptom prevalence.

We identified three clusters of patients with PCC, in which dominant persistent symptoms (i.e., those present in ≥50% of subjects) showed an additive pattern (Fig. 2, Table 4): Individuals in cluster A (40.8% of subjects) presented primarily with fatigue; those in cluster B (44.6% of subjects) had fatigue plus dyspnea, neurocognitive complaints, headache, myalgia, arthralgia, chest pain and tachycardia; individuals in cluster C (14.2% of subjects) had the same dominant symptoms of cluster B plus skin and smell alterations, dysphagia, diarrhea, and neurosensitive symptoms. The median number of symptoms per patient were 6 (IQR 3–10), 10 (IQR 8–13) and 14 (IQR 11–18) for clusters A, B, and C, respectively. Compared with subjects from clusters B and C, more subjects in cluster A were men and required hospitalization and ICU admission during acute COVID-19, whereas fewer of them had a previous history of allergy.

**Table 4:** Characteristics of the post-COVID-19 condition clusters.

	Cluster A	Cluster B	Cluster C
N (%)	139 (40.8)	152 (44.6)	50 (14.2)
Age, years, median (IQR)	50 (42–57)	47 (38–56)	45 (39–51)
Sex, female, N (%)	73 (52.5)	120 (78.9)	45 (90)
Hospitalization, N (%)	65 (46.8)	52 (34.2)	13 (26)
Intensive care, N (%)	10 (7.2)	5 (3.3)	1 (2)
Comorbidities, N (%)			
Allergy	31 (22.3)	56 (36.8)	20 (40)
Obesity	32 (23.0)	39 (25.7)	13 (26.0)
Dyslipidemia	30 (21.6)	43 (28.3)	9 (18.0)
Hypertension	34 (24.5)	25 (16.4)	8 (16.0)
Lung disease	21 (15.1)	31 (20.4)	6 (12.0)
Persistent symptoms, N (%)			
Fatigue	100 (71.9)	139 (91.4)	48 (96.0)
Neurocognitive complaints	62 (44.6)	128 (84.2)	46 (92.0)
Dyspnea	45 (32.4)	131 (86.2)	44 (88.0)
Headache	41 (29.5)	103 (67.8)	44 (88)
Myalgia	31 (22.3)	98 (64.5)	33 (66)
Arthralgia	39 (28.1)	92 (60.5)	47 (94)
Chest pain	31 (22.3)	76 (50.0)	45 (90)
Tachycardia	19 (13.7)	83 (54.6)	39 (78.0)
Cough	18 (12.9)	64 (42.1)	19 (38.0)
Neurosensitive symptoms	31 (22.3)	66 (43.4)	40 (80.0)
Diarrhea	14 (10.1)	59 (38.8)	26 (52.0)
Low grade fever	13 (9.35)	36 (23.7)	21 (42.0)
Smell alterations	34 (24.5)	38 (25)	30 (60)
Dermatological alterations	24 (17.3)	36 (23.7)	43 (86)
Dysphagia	9 (6.47)	18 (11.8)	27 (54)
Dysphonia	10 (7.19)	29 (19.1)	19 (38)
Recovery from PCC, N (%)	24 (17.3)	1 (0.7)	1 (2.0)

Of the 152 individuals reporting chest pain, 36 (23.7%) fulfilled anginal characteristics; 22 of them (61.1%) underwent adenosine-stress MRI because of suspected angina, revealing myocardial perfusion defects with normal coronary arteries in 9/22 individuals (41.0%) and myocarditis in one subject (Supplementary Figure S3). The remaining 14 subjects had contraindications or did not consent to be tested. Cardiac MRI-confirmed perfusion abnormalities were more frequent among individuals in cluster C (5/50 individuals; 10%) than in clusters B (4/142 subjects; 2.6%) and A (zero subjects, 0%).

### Recovery from the PCC

Only 26 (7.6%) of study participants recovered from the PCC during follow-up. The median time to recovery among these subjects was 11.4 months (IQR 6.1–13.3). Almost all of them (n = 24) belonged to cluster A; only one individual belonged to each cluster B and C.

**Fig. 3:**
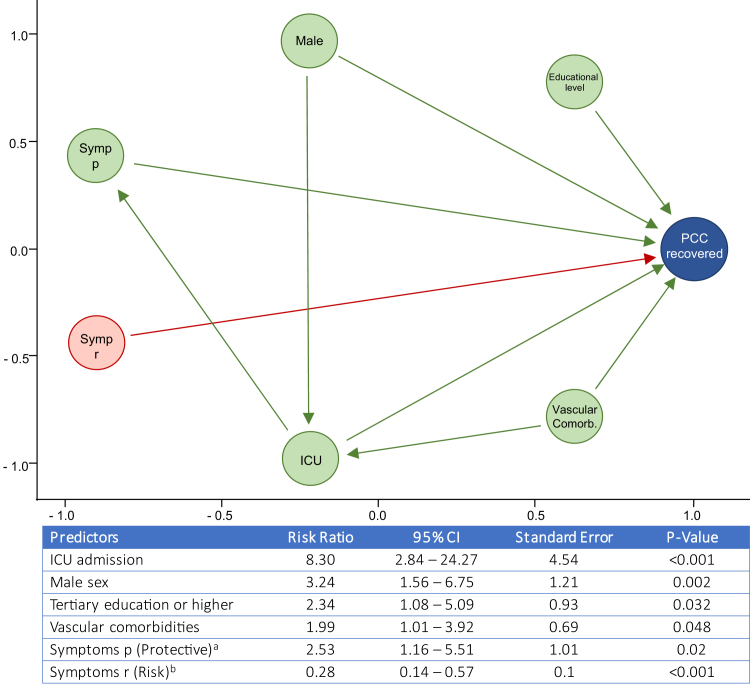
**Model of recovery from the post-COVID-19 condition.** Directed acyclic graph of causal relationships between factors present during the acute COVID-19 episode and their influence on the chance of recovery from the PCC. Green arrows indicate positive associations; red arrows show negative associations. The statistical model metrics are shown below the graph. ICU indicates intensive care unit. Symp p (protective) indicates symptoms developed during acute COVID-19 which are associated with recovery from PCC, i.e., hyporexia and smell and taste alterations. Symp r (risk) indicates symptoms developed during acute COVID-19 which are associated with lack of recovery from PCC, i.e., myalgia, dyspnea, tachycardia, neurocognitive disorder. Educational level indicates tertiary education or higher. Vascular comorbidities include hypertension, diabetes and dyslipidemia.

The directed acyclic graph causal model for PCC recovery (Fig. 3) indicated that male sex (Risk ratio, RR = 3.24, 95% CI: 1.56–6.75), prior ICU admission (RR = 8.3, 95% CI: 2.84–24.27), a tertiary educational level (RR = 2.34, 95% CI: 1.08–5.09) and the presence of cardiovascular comorbidities (hypertension, diabetes and/or dyslipidemia) during the acute COVID-19 episode (RR = 1.99, 95% CI: 1.01–3.92) were associated with a higher likelihood of subsequent recovery from the PCC. Importantly, subjects with smell and taste alterations and hyporexia during the acute COVID-19 episode were also more likely to recover from the PCC thereafter (RR = 2.53, 95% CI: 1.16–5E51). Conversely, the presence of muscle pain, impaired attention, dyspnea, or tachycardia during acute COVID-19 was associated with a reduced likeliness of recovering from the PCC (RR = 0.28, 95% CI: 0.14–0.57).

### SARS-CoV-2 vaccination and PCC symptoms

**Fig. 4:**
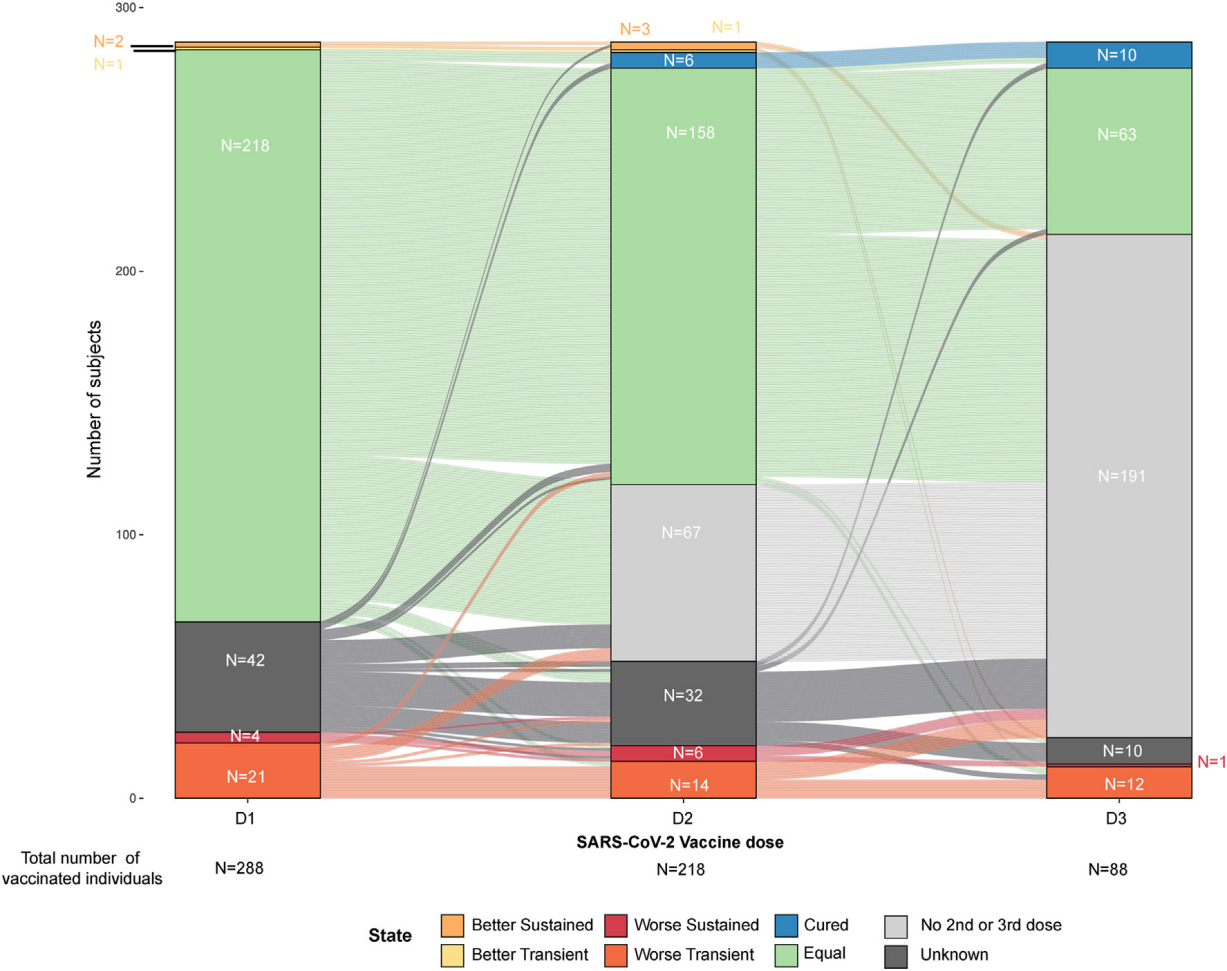
**Post-COVID-19 condition symptoms and SARS-CoV-2 vaccination.** Stacked bar plot of overall symptom status reported by subjects with PCC one month after each SARS-CoV-2 vaccine dose administration, relative to their symptom status during the month prior to receiving the corresponding vaccine dose. Subjects’ reports belonged to 6 qualitative categories: (i) Equal: no changes in symptoms (green); (ii) Better, sustained: sustained improvement during the month following the vaccine dose (light orange); (iii) Better, transient: transient improvement following the vaccine dose with return to the pre-dose status by the end of the month (yellow); (iv) Worse, sustained: sustained worsening during the month following the vaccine dose (red); (v) Worse, transient: transient improvement following the vaccine dose with return to the pre-dose status by the end of the month (dark orange). Subjects with remission of all PCC symptoms (Cured) are shown in blue, unknown values in dark grey and no vaccine doses in light grey.

Of all individuals with PCC (n = 341), 13 (3.8%) subjects with PCC recovered before receiving the first vaccine dose, 40 (11.7%) did not receive any immunization, and 288 (84.4%) received at least one dose of SARS-CoV-2 vaccine: 218/341 (63.9%) received two doses, and 88/341 (25.8%) three doses. Most of the individuals with PCC and at least one vaccine dose (217/288; 75.3%) did not experience any change in persistent symptoms after first vaccine dose (Fig. 4). Only 3 participants reported an improvement of symptoms after the first immunization, while 25 PCC patients described a worsening of PCC symptoms matching immunization (transient in 21 and sustained in 4). Most individuals who reported worsening of symptoms after the 1st vaccine dose, also felt worse after the following immunizations (Fig. 4).

## Discussion

In this 2-year, prospective, systematic assessment of a large Southern European hospital cohort of COVID-19 survivors, we found that preexisting medical conditions, including several comorbidities, socioeconomic factors like the educational level, and specific symptoms presenting during acute COVID-19 onset, predicted both the development of and recovery from the PCC. In concordance with the US RECOVER cohort,^13^ individuals presented with sub-syndromic clusters characterized by accumulation of overlapping symptoms rather than by mutually excluding syndromic profiles. Worryingly, recovery from the PCC was rare during the first two years, which poses a major challenge to European healthcare systems. As long as SARS-CoV-2 transmission continues and few people are cured from the PCC, subjects with such disabling post-viral syndrome will continue to accumulate and will have to be properly absorbed and managed by currently unprepared systems.

Emerging pathophysiological understanding of the PCC shows that SARS-CoV-2 infection triggers a wide and diverse variety of insults of different molecular nature (endotheliopathy, thrombosis, hemorrhage, autoimmunity, immune activation, inflammation, microbial dysbiosis, etc.) at various cellular, tissular, organic and systemic levels, with a considerable interindividual variation.^23^ Viral persistence, either from viral remnants or reservoirs, could contribute to these effects.^24^^,^^25^ Viral RNA and/or antigens have been found not only in individuals who died from acute disease, but also in infected individuals weeks to months after asymptomatic or mild infection with SARS-CoV-2.^24^, ^25^, ^26^ Hence the ongoing debate as to whether the PCC is a single entity or, instead, a heterogeneous composite of subsyndromes with an independent pathophysiological basis. The pattern of the observed subphenotypes in our study most likely suggests additive severity of a single, multisystemic, multifaceted post-viral disease rather than different pathogenically-independent subsyndromes. This is important as the current challenge lies in accurately identifying patient profiles who could benefit from tailored treatment approaches. Our findings are in line with previous studies, which clustered patients according to symptom distribution at a given time point, and consistently identified clusters with increasing severity and an additive symptom pattern.^13^^,^^27^, ^28^, ^29^, ^30^ In contrast, one study by Davis et al.^5^ identified three clusters with mutually-exclusive onset patterns of symptoms using a time-course clustering approach.

Cardiovascular and/or cognitive disorders were particularly prevalent in at least one of the clusters. Symptoms like fatigue and dyspnea were highly prevalent across the three clusters, whereas others like tachycardia or cough had a high prevalence only in cluster C, which also showed a high prevalence of other symptoms. Interestingly, chest pain, identified in approximately 45% of individuals in clusters B and C, correlated with heart abnormalities on MRI in a significant proportion of these patients. The frequency of MRI-confirmed perfusion abnormalities was higher among individuals in cluster C (5/50 individuals; 10%) than in the other clusters: 2.6% (4/142) in cluster B and 0 in cluster A. Although limited in number, these findings add to previous knowledge indicating cardiovascular damage in the PCC.^31^

Our findings and, in our opinion, those from the RECOVER and other studies, show that, although statistically, syndromic clusters can often be identified among PCC patients, clinically, symptom overlap and individual variability are the rule. The observed clusters in all studies are largely driven by a discrete set of dominant symptoms that show a cumulative pattern and frequently correlate among themselves. This implies that the number and specific composition of the PCC clusters will be highly dependent on the characteristics (i.e., size and nature) of the patient group analyzed. As a consequence, specific symptom clustering is unlikely to be sufficiently consistent and reproducible across cohorts to become useful for daily clinical management or reliable clinical trial design. It is thus urgent to advance our knowledge to identify reliable, objective biomarkers of the PCC.

One important finding of our study was that the initial COVID-19 clinical presentation, along with the patient's comorbidity background and educational level, were associated with both the risk of onset of and recovery from the PCC. A plethora of symptoms shown in Fig. 1 were highly associated with PCC and a model including several preexisting conditions as well as symptoms at COVID-19 onset achieved a remarkable ability to discriminate between subjects with and without PCC. Preexisting conditions associated with PCC onset (i.e., female sex and previous history of autoimmunity, arrhythmia, and allergy) among subjects with PCC suggest a certain degree of host predisposition to at least some of the PCC features. This requires proper further investigation on risk factors that includes large GWAS and innate immunity studies in both, adults and children, for which risk factors may differ.^32^

A novel finding of our study was that individuals with tertiary education or higher were less likely to suffer and more likely to recover from PCC. Further studies are warranted to disentangle the relative contribution of higher cognitive reserve and socioeconomic status in the development and evolution of the PCC.

Worryingly, we found that despite the large follow-up time of our study (median 23 months, the largest so far), recovery from PCC was exceptionally rare in our cohort: 8% overall and 13% for the most favored cluster. These figures are worse than those reported by previous observational studies. The PHOSP-COVID study, which followed a cohort of PCC patients hospitalized during the acute episode, reported a 29% recovery rate at one year,^28^ whereas survey-based studies reported even higher recovery rates (i.e., up to 35%) after shorter follow-up periods.^5^^,^^7^ These conflicting figures might be partially explained by differences in patient characteristics (i.e., post-discharge patients in the PHOSP-COVID cohort vs. mixed severity in ours) or reporting bias potentially associated with survey-based studies. A strength of our study relative to others is that our symptom collection strategy was systematic, prospective, and supervised by the expert clinical team, which minimizes symptom underreporting. Subjects with PCC may experience fluctuating symptoms with relapse-remission cycles; therefore, short follow-up periods or long time lapses between assessments may overestimate the apparent recovery rate from PCC. Regardless of the exact cure rates, the long-lasting persistence of PCC in most patients highlights the clinical and public health importance of this condition, which globally might have a remarkable impact on the number of years with disability.

One of the unmet needs in PCC science is, precisely, the identification of factors that may influence the likelihood of recovery. Owing to the extremely low number of individuals who recovered from PCC in our cohort (also observed in other large observational studies),^28^ we used a DAG approach^18^^,^^33^ to investigate relationships between some factors and the likelihood of recovery. Although the results of this analysis must be seen as exploratory, patients with involvement of the central nervous and cardiovascular systems during the acute COVID-19 episode were less likely to recover. Importantly, ICU admission during the acute COVID-19 episode showed the highest adjusted risk ratio for recovery. This result was conflicting with the PHOSP-COVID study, which identified the need for invasive mechanical ventilation among the three most important risk factors for lack of recovery (along with obesity and sex).^28^ Such inconsistency must be interpreted with caution: all patients in the PHOSP-COVID study had been hospitalized during the acute episode, whereas in our analysis, 62% of participants did not require hospitalization. ‘Post Acute COVID-19 Sequelae (PACS)’ is a less restrictive concept than PCC, as currently defined by the WHO, and may include subjects with ‘Post-Intensive Care Syndrome (PICS)’. The latter has been known for decades and has a different pathogenesis, mainly related to sequelae from severe sepsis and systemic inflammation, prolonged immobilization, and exposure to invasive medical interventions. Although PACS, PCC, and PICS often overlap syndromically, the prognosis of PICS is better than that of PACS or PCC, which is also reflected in our own models for PCC recovery.

The impact of vaccination in persistent symptomatology is controversial.^34^ In our cohort, most participants did not report symptom changes after SARS-CoV-2 vaccine administration. Only 21 and 4 PCC participants experienced a transient and sustained worsening of persistent symptoms, respectively, whereas PCC recovery, although rare, also increased over time. Importantly, our study design does not allow to establish any causal relationship between SARS-CoV-2 vaccination and PCC symptoms, and should be interpreted strictly as a descriptive exercise. We cannot rule out that any modification of symptoms could be related to the relapsing nature of the PCC, or to any other confounding factor.

Although this study is one of the largest systematic evaluations of PCC clinical evolution available to date, it also has several limitations. The study cohort was created early in the pandemic, when PCC was not fully recognized; therefore, selection bias favoring the inclusion of the most severe cases cannot be fully ruled out. Likewise, this was a hospital cohort, which can contribute to this same bias. Collection of symptoms in a dichotomized manner (i.e., presence/absence of each symptom) with no gradation as to the specific impact of each and any of them on daily activities or quality of life might also contribute to inflate the overall severity of the clinical picture described. Of note, all subjects were first infected during the pre-Omicron era, and almost none of them had been previously vaccinated against SARS-CoV-2, encouraging carefulness when extrapolating our findings to current incident PCC cases. As mentioned previously, no causal relationship between SARS-CoV-2 vaccination and PCC clinical evolution should be extracted from this work. Similarly, associations between clinical and socioeconomic factors and the longitudinal evolution of PCC should not be interpreted as causal. The low number of subjects recovered from PCC warrants particular caution when interpreting our DAG models. Despite these limitations, our study evaded important limitations of studies conducted so far, mostly based on surveys or patient self-reports. In this regard, the prospective, face-to-face assessment of PCC provides a more accurate view of the clinical characterization of this condition.

In summary, this study shows that the initial COVID-19 clinical presentation, along with the comorbidity background and educational level of the patient, are useful in predicting both the risk of onset of and recovery from the PCC. Unfortunately, the small chances of recovering from PCC during the first two years underscore that, as long as SARS-CoV-2 transmissions continue and few people are cured, subjects with PCC will continue to accumulate. European healthcare systems must be prepared to absorb and manage such demand. Novel objective biomarkers, deeper pathophysiological insights, and innovative therapies are urgently needed to prevent and cure PCC or, at least, mitigate its effects and its public health impact.

## Contributors

The study was designed and conceived by LM, RP, and MM. LM, CL, JRS, GL, CL, SE-C, RT, MF, AC, MN, NV, AT, CR-F, JAM-M, AP, CE, RC, CH, PC, AG, RP, and MM contributed to data collection. The analysis was conducted by LM, CT, FM-L, CH, PC, AG, RP, and MM, and the results interpreted by LM, CT, CL, JRS, GL, CrL, SE-C, FM-L, RP, BC, and MM. The manuscript was first drafted by LM, CT, RP, and MM. LM, CL, CT, JRS, GL, CrL, SE-C, RT, MF, AC, FM-L, MN, NV, AT, JP, CR-F, JAM-M, AP, CE, RC, CH, PC, AG, RP, BC, and MM made substantial contributions to revising the successive drafts and approved the final version of the manuscript.

## Data sharing statement

Clinical phenotype data from this study are held by the data management team, and can be made available to other research groups, after approval of a proposal and with a signed data access agreement. Please contact the corresponding author with an outline of the intended use.

## Declaration of interests

LM has received grants from Grifols, honoraria as speaker from Astra-Zeneca, Gilead, and Pfizer, and has participated in advisory boards for Gilead and MSD. CL has received support for attending meetings from Gilead. AG has received grants from Grifols, honoraria for lectures or presentations from Astra-Zeneca, Gilead, and Pfizer, and has participated on DSMB or advisory boards for Gilead and MSD. RP has participated in advisory boards for Pfizer, Gilead, MSD, GSK, Atea, Lilly, Roche, Astra-Zeneca, ViiV Healthcare and Theratechnologies, has participated in lectures and seminars funded by Gilead, Pfizer, GSK and AstraZeneca, and has received research funds awarded to his institution from Gilead, Pfizer, and MSD. MM has received honoraria for participating in lectures and seminars funded by Gilead. MM was granted with RYC2020-028934-I/AEI/10.13039/501100011033 from Spanish Ministry of Science and Innovation and State Research Agency and the European Social Fund “investing in your future”. This work had the additional collaboration of “Programa de Becas Gilead a la Investigación Biomédica, GLD21_00070”. FM was supported by Sorigué Foundation. The other authors declare no competing interests.

## Appendix A Supplementary data

Supplementary Appendix

Translated Abstract Catalan

Translated Abstract Spanish
