# Supplementary material for: Determinants of the onset and prognosis of the post-COVID-19 condition: a 2-year prospective observational cohort study
Source: Lancet Reg Health Eur. 2023 Sep 5;33:100724. doi: 10.1016/j.lanepe.2023.100724 (PMC10636281; doi:10.1016/j.lanepe.2023.100724)
Supplement: Supplementary Appendix [file mmc1.docx]

**Supplementary Appendix**

Supplement to:

Mateu L, et al: Determinants of the onset and prognosis of the Post-COVID-19 Condition: a 2-year prospective observational cohort study

This appendix has been provided by the authors to give readers additional information about the work.

**Supplementary Results**

**Table S1: Comorbidities at study entry**

|  | **PCC**  **N=341 (%)** | **No PCC**  **N=207 (%)** |
| --- | --- | --- |
| Smoking status (current or past) |  |  |
| Current smoker | 30 (9.17) | 13 (8.7) |
| Former smoker | 120 (36.7) | 61 (40.9) |
| Never smoked | 177 (54.1) | 75 (50.3) |
| Menopause* | 84 (35.3) | 22 (21.2) |
| Allergy (drug, food or environmental) | 107 (31.4) | 38 (18.4) |
| Alcohol consumption | 103 (30.2) | 61 (41.2) |
| Obesity | 84 (24.6) | 29 (14.0) |
| Dyslipidemia | 82 (24.0) | 59 (28.5) |
| Arterial Hypertension | 67 (19.6) | 45 (21.7) |
| Headache | 66 (19.4) | 10 (4.8) |
| Lung disease | 58 (17.0) | 27 (13.0) |
| Insomnia | 39 (11.4) | 7 (3.4) |
| Autoimmune disease | 37 (10.9) | 9 (4.3) |
| Gastrointestinal disease | 37 (10.9) | 15 (7.2) |
| Cardiac arrhythmias | 33 (9.7) | 9 (4.3) |
| Asthma | 24 (7.0) | 9 (4.3) |
| Liver disease | 24 (7.0) | 11 (5.3) |
| Solid neoplasia | 22 (6.4) | 19 (9.2) |
| Post-viral fatigue syndrome | 22 (6.4) | 1 (0.5) |
| Diabetes mellitus | 21 (6.2) | 26 (12.6) |
| Chronic Fatigue Syndrome | 16 (4.7) | 0 (0.0) |
| Fibromyalgia | 14 (4.1) | 1 (0.5) |
| Thromboembolic disease | 7 (2.0) | 3 (1.4) |
| Ischemic heart disease | 6 (1.8) | 7 (3.4) |
| Kidney failure | 6 (1.8) | 8 (3.9) |
| Immunosuppressive therapy | 6 (1.8) | 10 (4.8) |
| COPD | 5 (1.4) | 6 (2.9) |
| Stroke | 3 (0.9) | 3 (1.4) |
| HIV-1 infection | 2 (0.6) | 15 (7.2) |
| Primary immunodeficiency | 2 (0.6) | 1 (0.5) |
| Solid organ transplantation | 2 (0.6) | 3 (1.4) |
| Hematological neoplasia | 1 (0.3) | 5 (2.4) |
| Heart failure | 1 (0.3) | 1 (0.5) |

PCC, Post-COVID-19 Condition; COPD, Chronic Obstructive Pulmonary Disease

* Refers only to the 93 and 238 women without and with PCC, respectively, included in the study

**Figure S1. Study flow-chart**

**Figure S2. Prevalence of persistent symptoms**

^a^ Note: Refers to the whole study cohort; HEENT: Head, Eyes, Ears, Nose & Throat

**Figure S3: Myocardial hypoperfusion in a stress cardiac magnetic resonance (CMR).**

**Panel A,** normal myocardial volumes and ejection fraction; **Panel B,** absence of diffuse myocardial fibrosis (normal T1 mapping values); **Panel C,** absence of myocardial edema (normal T2 mapping values; panel C); **Panel D**, circumferential inducible perfusion defects during adenosine stress perfusion (white arrows), and **Panel E**, absence of late gadolinium enhancement.


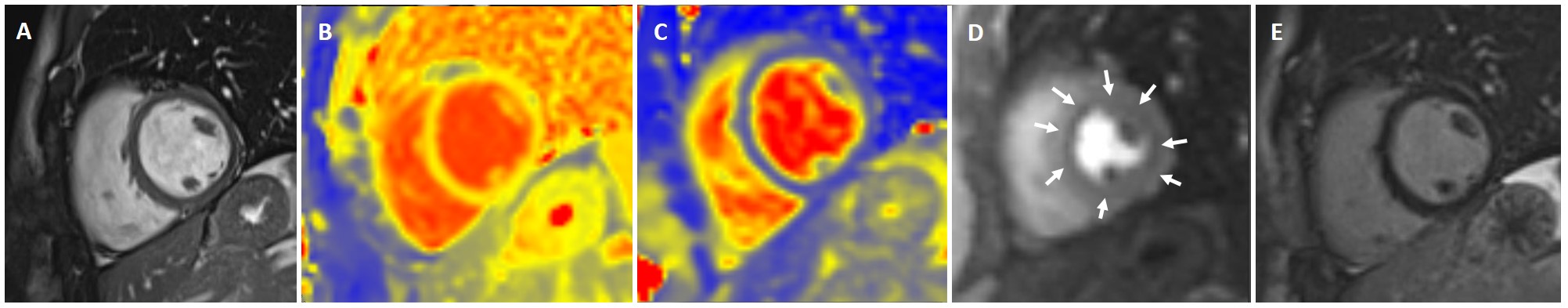


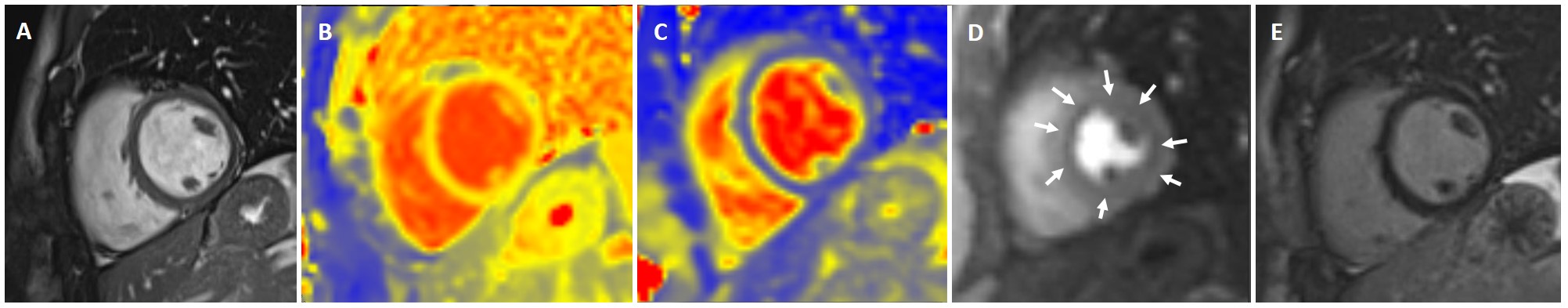


**Questionnaires**

| **GERMANS TRIAS´ LONG COVID SYMPTOM QUESTIONNAIRE** | | | |
| --- | --- | --- | --- |
| ***Symptom*** |  | **Points** |  |
| **Fever >38ºC** |  | +5 |  |
| **Low grade fever 37-38ºC** |  | +4 |  |
| **Dysthermia sensation** |  | +3 |  |
| **Fatigue / Asthenia**   | No fatigue | +0 |  |
|  | Mild fatigue | +1 |  |
|  | Moderate fatigue | +2 |  |
|  | Severe fatigue | +3 |  |
|  | Very severe fatigue | +4 |  |
|  | The worst fatigue | +5 |  |
| **Shortness of Breath Grade** | Absence of dyspnea when performing intense exercise | +0 |  |
|  | None except during strenuous exercise | +1 |  |
|  | Occurring when hurrying on level ground or walking up a slight incline | +2 |  |
|  | Resulting in walking more slowly than people of the same age on level ground  Or  Resulting in stopping for breath when walking at own pace on level ground | +3 |  |
|  | Resulting in stopping for breath after walking about 100 meters or after a few minutes on level ground | +4 |  |
|  | Severe enough to prevent the person from leaving the house  Or  Occurring when dressing or undressing | +5 |  |
| **Cough** |  | +3 |  |
| **Expectoration** |  | +2 |  |
| **Chest pain**   | No pain | +0 |  |
|  | Mild pain | +1 |  |
|  | Moderate pain | +2 |  |
|  | Severe pain | +3 |  |
|  | Very severe pain | +4 |  |
|  | Worst pain possible | +5 |  |
| **Tachycardia (>100bpm)** |  | +3 |  |
| **Alterations in blood pressure control** |  | +2 |  |
| **Joint pain**   | No pain | +0 |  |
|  | Mild pain | +1 |  |
|  | Moderate pain | +2 |  |
|  | Severe pain | +3 |  |
|  | Very severe pain | +4 |  |
|  | Worst pain possible | +5 |  |

| **Muscle pain**   | No pain | +0 |  |
| --- | --- | --- | --- |
|  | Mild pain | +1 | |
|  | Moderate pain | +2 | |
|  | Severe pain | +3 | |
|  | Very severe pain | +4 | |
|  | Worst pain possible | +5 | |
| **Headache**   | No pain | +0 |  |
|  | Mild pain | +1 |  |
|  | Moderate pain | +2 |  |
|  | Severe pain | +3 |  |
|  | Very severe pain | +4 |  |
|  | Worst pain possible | +5 |  |
| **Sensitivity alterations (tingling)** |  | +3 |  |
| **Brain fog** |  | +3 |  |
| **Memory loss** |  | +2 |  |
| **Loss of attention/concentration** |  | +2 |  |
| **Difficulty planning** |  | +2 |  |
| **Dizziness** |  | +3 |  |
| **Taste alterations** |  | +2 |  |
| **Smell alterations** |  | +2 |  |
| **Alterations in the voice** |  | +2 |  |
| **Difficulty swallowing** |  | +2 |  |
| **Throat pain** |  | +2 |  |
| **Ear pain** |  | +2 |  |
| **Nausea** |  | +2 |  |
| **Abdominal pain**   | No pain | +0 |  |
|  | Mild pain | +1 |  |
|  | Moderate pain | +2 |  |
|  | Severe pain | +3 |  |
|  | Very severe pain | +4 |  |
|  | Worst pain possible | +5 |  |
| **Diarrhea** |  | +3 |  |
| **Decrease in food intake** |  | +2 |  |
| **Weight loss** |  | +2 |  |
| **Hair loss** |  | +2 |  |
| **Dry eyes and mouth** |  | +2 |  |
| **Skin disorders** |  | +2 |  |
| **Alterations in menstruation** |  | +2 |  |
| **Tinnitus** |  | +2 |  |
| **Vision alterations** |  | +2 |  |
|  | **TOTAL :** |  |  |

Source: Germans Trias Long COVID Unit

| **FATIGUE SEVERITY SCALE (FSS)** | |
| --- | --- |
|  | **SCORES** |
|  | **1=Strongly disagree; 7 = Strongly agree** |
| 1. **My motivation is lower when I am fatigued** | **1 2 3 4 5 6 7** |
| 1. **Exercise brings on my fatigue** | **1 2 3 4 5 6 7** |
| 1. **I am easily fatigued** | **1 2 3 4 5 6 7** |
| 1. **Fatigue interferes with my physical functioning** | **1 2 3 4 5 6 7** |
| 1. **Fatigue causes frequent problems for me** | **1 2 3 4 5 6 7** |
| 1. **My fatigue prevents sustained physical functioning** | **1 2 3 4 5 6 7** |
| 1. **Fatigue interferes with carrying out certain duties and responsibilities** | **1 2 3 4 5 6 7** |
| 1. **Fatigue is among my three most disabling symptoms** | **1 2 3 4 5 6 7** |
| 1. **Fatigue interferes with my work, family, or social life** | **1 2 3 4 5 6 7** |

Source: Krupp LB, LaRocca NG, Muir-Nash J, Steinberg AD. The fatigue severity scale. Application to patients with multiple sclerosis and systemic lupus erythematosus. Arch Neurol. 1989 Oct;46(10):1121-3. doi: 10.1001/archneur.1989.00520460115022. PMID: 2803071.

| **GERMANS TRIAS´ LONG COVID PATIENT CHARACTERISTICS QUESTIONNAIRE** | |
| --- | --- |
| Age |  |
| Ethnicity (origin /country) |  |
| Profession |  |
| Health worker (yes/no) |  |
| Level of education | |
|  | **Less than basic education** ( no schooling, early childhood education) |
|  | **Primary education** (primary education or first stage of basic education) |
|  | **Intermediate education** (secondary education) |
|  | **Advanced education** (bachelor’s or equivalent level) |
| Cigarette smoking | |
|  | Never smoker |
|  | Current smoker (cigarettes/ day and years smoking) |
|  | Former smoker (cigarettes/ day and years smoking) |
| Alcohol (UBEs/day) |  |
| Physical exercise (hours/week) |  |
| Comorbidities |  |
|  | Allergies |
|  | Drug |
|  | Environmental |
|  | Alimentary |
|  | Hypertension |
|  | Diabetes mellitus |
|  | Obesity |
|  | Dyslipidemia |
|  | Asthma |
|  | COPD |
|  | Renal failure |
|  | Cancer |
|  | Immunosuppressive treatment |
|  | Biological therapy |
|  | HIV |
|  | Primary immunodeficiency |
|  | Other |
| Acute SARS-CoV-2 infection |  |
|  | Date of symptom onset |
|  | Symptoms |
|  | Fever |
|  | Arthralgia |
|  | Myalgia |
|  | Diarrhea |
|  | Nausea |
|  | Cough |
|  | Dyspnea |
|  | Odynophagia |
|  | Smell alteration |
|  | Taste alteration |
|  | Tachycardia |
|  | Headache |
|  | Others |
|  | Admission to a critical care unit |
|  | Semi critical-care unit |
|  | Intensive care unit |
|  | Oxygen required |
|  | None |
|  | Nasal cannula |
|  | Venturi mask |
|  | Monaghan |
|  | High-flow nasal cannula |
|  | Non-mechanical ventilation |
|  | Mechanical ventilation |
|  | Extracorporeal membrane oxygenation |
|  | Diagnostic method ( and data) |
|  | PCR |
|  | RAT |
|  | Serology |
|  | Non |
| Height (cm) |  |
| Weight (kg) |  |
| Body mass index (BMI) |  |
| Dynamometer |  |
| Dominant hand |  |
| Measure 1 (kg) |  |
| Measure 2 (Kg) |  |
| Measure 3 (kg) |  |
| Bioimpedance |  |
| Performed ( yes/no) |  |

Source: Germans Trias Long COVID Unit
