# Supplementary material for: Determinants of the onset and prognosis of the post-COVID-19 condition: a 2-year prospective observational cohort study
Source: Lancet Reg Health Eur. 2023 Sep 5;33:100724. doi: 10.1016/j.lanepe.2023.100724 (PMC10636281; doi:10.1016/j.lanepe.2023.100724)
Supplement: Translated Abstract Catalan [file mmc2.docx]

*This translation in Catalan was submitted by the authors and we reproduce it as supplied. It has not been peer reviewed. Our editorial processes have only been applied to the original abstract in English, which should serve as reference for this manuscript.*

**Resum**

**Antecedents:** Com a mínim, el 5-10% de les persones que sobreviuen a la COVID-19 desenvolupen la Condició Post-COVID-19 (CPC) o “Long COVID”. La presentació clínica de la CPC és heterogènia, la seva patogènesi s’està desxifrant, i manquen biomarcadors objectius validats. Es desconeix si la CPC és una entitat única o una síndrome heterogènia amb bases fisiopatològiques solapades. L’estudi americà RECOVER va identificar quatre grups de pacients amb CPC d’acord als símptomes que presentaven. No obstant, es desconeixen les implicacions de la CPC a llarg termini.

**Mètodes:** Vàrem realitzar un estudi de cohorts prospectiu de 2 anys de durada amb persones que sobrevivien la COVID-19, incloent persones que complien la definició de CPC de la OMS i persones amb recuperació completa. Vam recollir de forma sistemàtica els símptomes post-COVID utilitzant qüestionaris pre-definits i vam realitzar estudis diagnòstics d’imatge addicionals quan va ser necessari. Vam identificar i modelitzar els factors associats amb la CPC mitjançant regressió logística. Es va utilitzar una anàlisi d’agrupació no supervisada per agrupar els pacients amb CPC d’acord als símptomes que presentaven. Es van modelitzar els factors associats amb recuperació de la CPC utilitzant una tècnica gràfica acíclica directa.

**Troballes:** L’estudi va incloure 548 persones, 341 amb CPC, seguides durant una mediana de 23 mesos (IQR 16·5 – 23·5), i 207 persones completament recuperades. En el model amb millor ajust, els homes i les persones amb estudis terciaris van tenir menys risc de desenvolupar CPC; en canvi, tenir antecedents de mal de cap o presentar taquicàrdia, fatiga, queixes neurocognitives o neurosensitives i dispnea al diagnòstic de COVID-19 va predir el desenvolupament de CPC. L’anàlisi d’agrupació de símptomes va identificar 3 perfils de pacients amb un nombre additiu de símptomes. Només 26 persones (7·6%) es varen recuperar de la CPC durant el seguiment; gairebé totes elles (n=24) pertanyien al grup A, menys simptomàtic i dominat principalment per fatiga. La recuperació de la CPC fou més probable als homes i les persones que havien requerit ingrés a cures intensives o bé tenien comorbiditats cardiovasculars, hiporèxia o alteracions a l’olfacte o el gust durant la COVID-19 aguda. Les persones que presentaven dolors musculars, disminució a la capacitat d’atenció, díspnea o taquicàrdia, en canvi, va ser menys probable que es recuperessin de la CPC.

**Interpretació:** Factors mèdics i socieconòmics pre-existents, així com alguns símptomes de COVID-19 aguda, s’associen al desenvolupament i recuperació de la CPC. La recuperació és extraordinàriament infreqüent durant els primers 2 anys, el que suposa un gran repte pels sistemes de salut.

**Finançament**: Fundació Lluita contra les Infeccions
