# Supplementary material for: Determinants of the onset and prognosis of the post-COVID-19 condition: a 2-year prospective observational cohort study
Source: Lancet Reg Health Eur. 2023 Sep 5;33:100724. doi: 10.1016/j.lanepe.2023.100724 (PMC10636281; doi:10.1016/j.lanepe.2023.100724)
Supplement: Translated Abstract Spanish [file mmc3.docx]

*This translation in Spanish was submitted by the authors and we reproduce it as supplied. It has not been peer reviewed. Our editorial processes have only been applied to the original abstract in English, which should serve as reference for this manuscript.*

**Resumen**

**Antecedentes**: Como mínimo, 5-10% de las personas que sobreviven en la COVID-19 desarrollan la Condición Post-COVID-19 (CPC) o “Long COVID”. La presentación clínica de la CPC es heterogénea, su patogénesis se está descifrando, y se carece de biomarcadores objetivos validados. Se desconoce si la CPC es una entidad única o un síndrome heterogéneo con bases fisiopatológicas solapadas. El estudio americano RECOVER identificó a cuatro grupos de pacientes con CPC de acuerdo a los síntomas que presentaban. Sin embargo, se desconocen las implicaciones de la CPC a largo plazo.

**Métodos**: Realizamos un estudio de cohortes prospectivo de 2 años de duración a personas que sobrevivieron la COVID-19, incluyendo personas que cumplían la definición de CPC de la OMS y personas con recuperación completa. Recogimos de forma sistemática los síntomas post-COVID utilizando cuestionarios predefinidos y realizamos estudios diagnósticos de imagen adicionales cuando fue necesario. Identificamos y modelizamos los factores asociados con la CPC mediante regresión logística. Se utilizó un análisis de agrupación no supervisado para agrupar a los pacientes con CPC de acuerdo a los síntomas que presentaban. Se modelizaron los factores asociados a la recuperación de la CPC utilizando una técnica gráfica acíclica directa.

**Hallazgos**: El estudio incluyó 548 personas, 341 con CPC, seguidas durante una mediana de 23 meses (IQR 16·5 – 23·5), y 207 personas completamente recuperadas. En el modelo con mejor ajuste, hombres y personas con estudios terciarios tuvieron menos riesgo de desarrollar CPC; en cambio, tener antecedentes de dolor de cabeza o presentar taquicardia, fatiga, quejas neurocognitivas o neurosensitivas y díspnea al diagnóstico de COVID-19, predijo el desarrollo de CPC. El análisis de agrupación de síntomas identificó 3 perfiles de pacientes con un número aditivo de síntomas. Sólo 26 personas (7-6%) se recuperaron de la CPC durante el seguimiento; casi todas ellas (n=24) pertenecían al grupo A, menos sintomático y dominado principalmente por fatiga. La recuperación de la CPC fue más probable en hombres y personas que habían requerido ingreso en cuidados intensivos o bien tenían comorbilidades cardiovasculares, hiporexia o alteraciones en el olfato o el gusto durante la COVID-19 aguda. Las personas que presentaban dolores musculares, disminución a la capacidad de atención, díspnea o taquicardia, en cambio, fue menos probable que se recuperaran de la CPC.

**Interpretación**: Factores médicos y socieconómicos preexistentes, así como algunos síntomas de COVID-19 aguda, se asocian al desarrollo y recuperación de la CPC. La recuperación es extraordinariamente infrecuente durante los primeros 2 años, lo que supone un gran reto para los sistemas de salud.

**Financiación**: Fundació Lluita contra les Infeccions
